# What are the benefits and harms of delayed enteral feeding in acute spinal cord injury patients in critical care units? A systematic review

**DOI:** 10.1177/17511437251328151

**Published:** 2025-03-25

**Authors:** Alex Gordon, Carla O’ Hagan, Jessie Welbourne

**Affiliations:** 1 Faculty of Health, Peninsula Medical School, University of Plymouth, Plymouth, UK; 2 Intensive Care Unit, University Hospitals Plymouth NHS Trust, Plymouth, UK

**Keywords:** Spinal cord injury, enteral nutrition

## Abstract

**Background:**

Spinal cord injury (SCI) is a devastating condition with a high burden of morbidity and mortality. National guidelines state that patients should not receive enteral feeding for 48 hours after inury, which may be detrimental if a patient experiences a hypercatabolic response to polytrauma. We conducted a systematic review of the benefits and harms of delayed enteral feeding in this population.

**Methods:**

We searched MEDLINE, EMBASE and Cochrane CENTRAL for studies which had a time parameter as part of their evaluation of feeding in the acute phase of spinal cord injury in a critical care setting. Required outcomes for inclusion were neurological improvement, neurological complications, time spent in an ICU, time to ICU discharge, incidence of secondary complications, other adverse effects and mortality. Risk of bias was assessed with the Downs and Black checklist.

**Results:**

Four studies met the inclusion criteria. There was no high-quality evidence of worsened outcomes with earlier feeding compared to delayed enteral feeding. One study demonstrated that patients fed before 24 h in conjunction with a broader bundle of care had improved neurological outcomes compared to previous non-standardised practice. There was no evidence of difference in frequency of infections or mortality in early or late feeding groups.

**Conclusions:**

We find no clear evidence of increased risk of harm from earlier enteral feeding strategies, nor clear evidence of benefit of earlier feeding as an isolated intervention.

## Introduction

Spinal cord injury (SCI) is a cause of life changing disability, with devastating consequences to patients and relatives alike. The annual incidence of SCI in the UK is estimated to be 19 cases per million,^1^ with an estimated mortality of up to 22.2%.^2^

Patients frequently need nutritional support in the acute phase of spinal cord injury. However, the possibility of ileus and/ or gastroparesis in the early stages post-injury may mean that delaying nutrition in SCI patients is potentially safer than administering enteral nutrition early on in a patient’s admission.^3,4^ This viewpoint is affirmed by the recommendation of the Multidisciplinary Association for Spinal Cord Injury Professionals (MASCIP), who recommend that the patient receives ‘nil enterally for the first 48 h if the individual is at risk of paralytic ileus’.^5^

Such an approach seems counterintuitive. This would not be the case in polytrauma cases without SCI due to the need to combat the acute hypercatabolic response,^6^ to avoid well documented sequelae of malnutrition in critically ill patients.^7^

The benefit of early enteral nutrition has been established in critically ill patients since the 1990s.^8^ A 2009 meta-analysis of non-neurological ICU patients validated these theoretical benefits, finding that, despite mechanistic reasoning about risk of ileus, pneumonia (including that secondary to aspiration) was in fact less likely in patients receiving enteral nutrition.^9^

International guidelines do not provide a consensus on when to initiate feeding either. The American Association of Neurological Surgeons identify diet as an important factor in the management of neurogenic bowel. Early and effective neurogenic bowel management is prioritised; however, no timeframe is stated to correlate with ‘early’ unlike the current European Society for Clinical Nutrition and Metabolism (ESPEN) guidelines which identify early as less than 48 h.^10^

As such, there is a lack of clarity on optimal timing to introduce nutrition in this patient population, and current guidelines in the UK appear to contradict international guidelines and evidence in similar patient populations. Therefore, we conducted a systematic review of the available published literature on feeding in the acute phase of SCI in critical care units to provide clarity on the benefits and risks of early versus delayed enteral feeding in this patient population.

## Methods

This review follows the guidelines set out in the PRISMA statement.^11^ The protocol was registered a priori on PROSPERO (CRD42023389016).

### Eligibility criteria

Participants had to have radiologically or clinically diagnosed acute spinal cord injury who were admitted to a critical care/ high dependency unit setting in the acute phase of their illness. Chronic spinal cord injury patients or patients in rehabilitation ward settings were excluded. Participants needed to receive enteral feeding with an explicit statement about time or range of times from admission or injury over which feeding was commenced. Studies where time parameters were not stated were excluded. We included studies that measured any clinical outcome including change in neurological status (e.g. motor function, sensory function), length of stay, complications secondary to spinal cord injury, other adverse effects, mortality and quality of life measures. This was for any available timepoints.

#### Participants/population

Patients admitted to an intensive care unit with an acute spinal cord injury. Acute spinal cord injury can be clinically or radiologically confirmed.

#### Intervention/exposure

Feeding which is initiated at a timepoint that is specified within studies

#### Comparator(s)/control

Feeding at a different timepoint or usual practice. Control is not a requirement for inclusion. Lack of a control group will not lead to exclusion.

Outcomes: Clinically relevant endpoints for the ICU clinician, rather than biochemical or metabolic parameters. We defined this as change in neurological status (e.g. motor function, sensory function) neurological complications, length of stay in ICU, length of stay in hospital, complications secondary to spinal cord injury, other adverse effects, mortality and quality of life measures.

There were no study design restrictions, however fully published manuscripts in peer-reviewed journals were required. Abstracts and poster presentations were excluded due to their brief format leading to inability to fully appraise methodology. Language was restricted to publications in English or where a translation of the original publication was available in English.

### Information sources

We searched Medline via Ovid, EMBASE via Ovid and the Cochrane CENTRAL trials database on the 12th February 2023 (Appendix 1). For studies that met inclusion after full text review, we also conducted forward and backward citation searching on papers which met full text inclusion criteria using Google Scholar.

### Search strategy

Our search strategy is outlined in Appendix 1.

### Selection process

**Figure 1.**
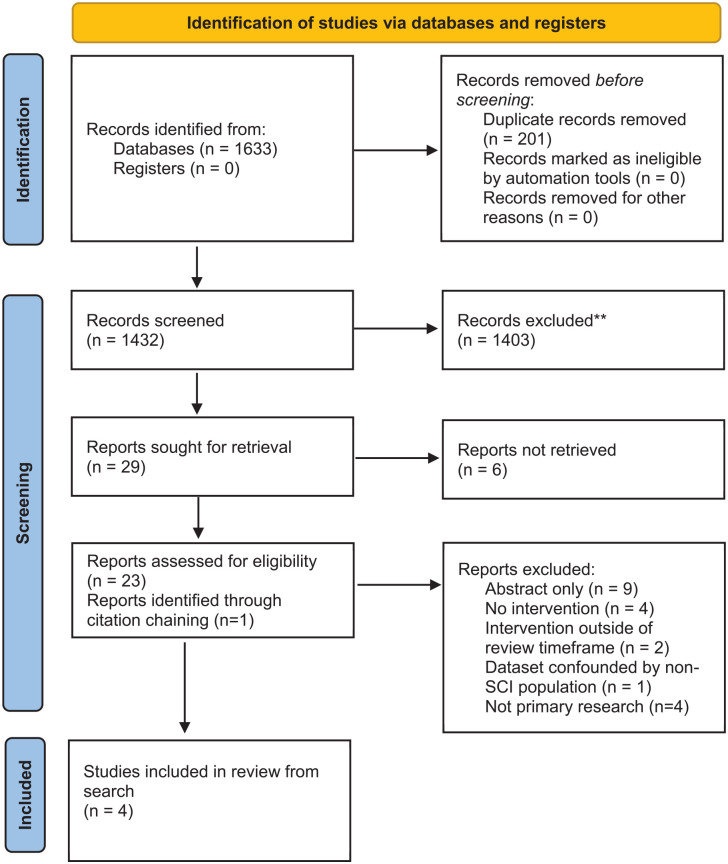
PRISMA flow diagram.

Two reviewers (AG and CO) screened all titles and abstracts independently through Rayyan. Disagreements between reviewers were resolved through discussion. The same process was used for full text review of eligible abstracts (See Figure 1).

### Data collection process

Data extraction was conducted independently by the same reviewers using a pre-prepared Google Form that was piloted on one of the studies to ensure it was able to extract the relevant variables. Differences in extraction were discussed and amended.

### Data Items

Participant characteristic data on level of spinal cord injury and modality of ascertaining this (radiological/clinical/both) and number of patients was extracted. We extracted data on nutrition requirement calculation, rate of feed, caloric content and constitution of enteral feed. We also looked at any confounding co-intervention.

### Study risk of bias assessment

Individual study risk of bias was assessed using the Downs and Black tool due to the heterogeneity of study design as this allows reasonable comparison between studies.^12^

### Effect measures

All effect measures are reported as they were in their original papers.

### Synthesis methods

Narrative synthesis was undertaken due to the methodological heterogeneity between studies.

## Results

### Study selection

The study selection process is detailed in Figure 1.

### Study characteristics

The four studies included in this review include data from 333 patients (Table 1). The studies were published between 1989 and 2019. One was based in France,^13^ two in North America,^14,15^ and one in New Zealand.^16^

**Table 1.** Study characteristics.

Authors	Location	Study design	Population	Intervention	Control	Outcomes	Funding
Cinotti et al. (2019)^13^	Nantes, France	Quasi-experimental pre-post	117 cervical SCI Patients	Bundle of care including enteral nutrition initiated in first 24h after ICU admission	Initiation of enteral nutrition performed according to guidelines and left to attending physician’s discretion	Primary: Change in neurological status (ASIA motor and sensory score) Secondary • Length of stay in ICU • Ventilatory parameters • Infection incidence • Ventilation duration • Noradrenaline duration • Mortality in ICU and at 1 year	None
Dvorak et al. (2004)^14^	Vancouver, Canada	Prospective RCT	17 SCI (C2-T1)	Enteral nutrition through NG tube before 72h after time of injury	Enteral nutrition through NG tube after 120h from time of injury	Primary: Septic complications Secondary • Length of stay in ICU • Duration of ventilation Feeding Complications	Foundation funds, no declared commercial partner
Kuric et al. (1989)^15^	Detroit, USA	Retrospective observational	166 quadriplegic/ paraplegic	Nutritional protocol: TPN from day 5 if not tolerating oral diet	Patients fed when clinically ‘ready’*	GI bleeding and/ or perforation	Not stated
Rowan et al. (2004)^16^	Auckland, New Zealand	Retrospective Observational	33 quadriplegic/ paraplegic	NG or NJ feed	None	Length of stay Enteral feed interruptions and rationale Mortality	Not stated

* Clinically ready was noted to convey a wide range of unprecise findings to multiple observers, although this was not specifically addressed enterally in the intervention group the study did find an earlier introduction of enteral feed.

Cinotti et al.^13^ conducted a pre-post quasi-experimental study evaluating multiple clinical outcomes in SCI patients given a bundle of care (including enteral feeding in the first 24 h) versus routine care.^13^ The RCT conducted by Dvorak et al.^14^ primarily examined difference in septic complications when patients received enteral nutrition before 72 h post-injury versus after 120 h post-injury.^14^ The earliest study, conducted by Kuric et al.^15^ in Detroit, USA examined gastrointestinal complications in 166 patients with a nutrition protocol that commenced feeding before 120 h if able versus feeding patients at clinician discretion.^15^ The fourth study, conducted by Rowan et al.^16^ in New Zealand, was a retrospective study which examined the effects of enteral feeding via nasogastric or nasojejunal feed on length of stay, mortality and enteral feed interruption.^16^

Patient and intervention characteristics from individual studies are shown in Table 2.

**Table 2.** Patient and intervention characteristics for studies.

Study	SCI level	Age	Sex (F:M)	Inclusion	Exclusion	Determination of SCI level	Feed nutrient calculation in intervention group	Feeding initiation in intervention group
Cinotti et al. (2019)^13^	Figure 3	Control = 45 ± 21 Intervention = 48 ± 23	Control = 20:37 Int = 16:44	Traumatic cervical SCI > 18yo	TBI (GCS < 12); admitted > 48 h after injury, non-traumatic SCI	Clinical limb impairment with CT or MRI confirmation	Regimen aims to achieve 25–30 kcal.kg by day 5	Within first 24 h of admission
Dvorak et al. (2004)^14^	Figure 4	Control = 41.4 ± 17.8 Int = 47.6 ± 16.8	Control = 1:6 Int = 1:9	ASIA grade A -C, last normal neurological level C2 – T1; admitted within 72h of injury	Pre-existing structural enteric disorder or malnutrition; additional injuries preventing NG feeding; major chest/ abdo trauma	Not reported	Predicted via Harris Benedict Equation with stress factor estimate up to 1.5	Before 72 h from time of injury
Kuric et al. (1989)^15^	Not reported	Control = 37; Int = 34.6	16:150	SCI patients between Jan 1982 & Jan 1985	Upper GI Tract pathology or prior injury to GI tract	Stratified by no injury vs. paraplegia vs quadriplegia	Calculated via Harris-Benedict Energy Equation	TPN given via central line after 4 days if no enteral feed tolerance
Rowan et al. (2004)^16^	Not reported	27* (15–47)	2:31	All SCI patients from Jan 1998 to 31 Dec 2000 with clinical spinal cord transection causing quadriplegia or paraplegia	Not specified	Not stated	standard, polymeric feed with a nutrient density of 1–1.2 kcal/m	2 days* (0.5–4.8)

* Median, else mean.

**Figure 2.**
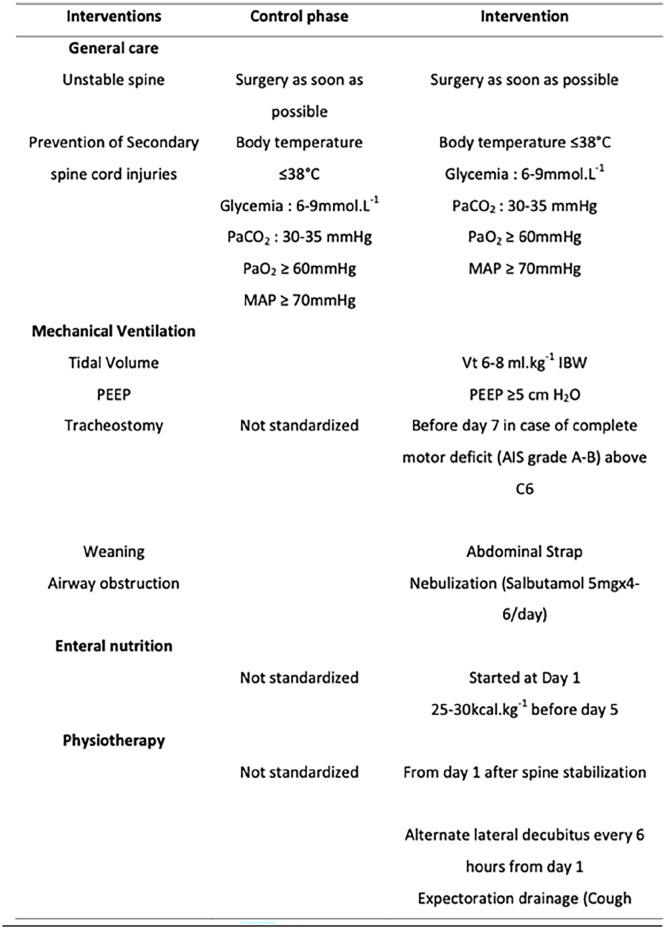
Bundle of interventions employed in the study by Cinotti et al. (2019).^13^

**Figure 3.**
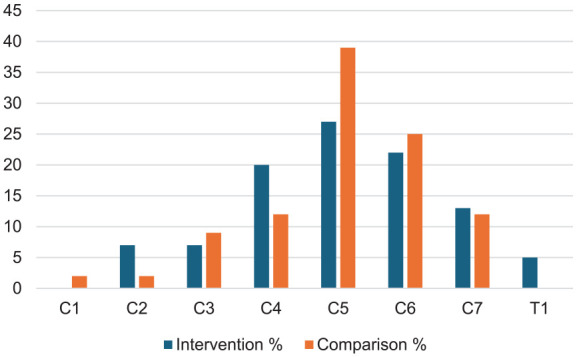
Distribution of SCI level across treatment groups in participants in the trial by Cinotti et al. (2019).^13^

Only Cinotti et al.^13^ and Dvorak et al.^14^ reported the specific injury level of their patients, with all being above the level of T1 (Figures 2 and 3).^13,14^ The average age of the patients ranged from 27 to 48 years old. About 83% of participants in studies were men. Only the Cinotti et al.^13^ reported using imaging to determine SCI level. Half of the studies report using the Haris Benedict Energy equation to calculate feeding requirement. In intervention groups, enteral feeding was started at a maximum of 24 h after admission up to 4.8 days from time of injury; Kuric et al.^15^ started total parenteral nutrition at 4 days if enteral feeding was not tolerated by this point.

**Figure 4.**
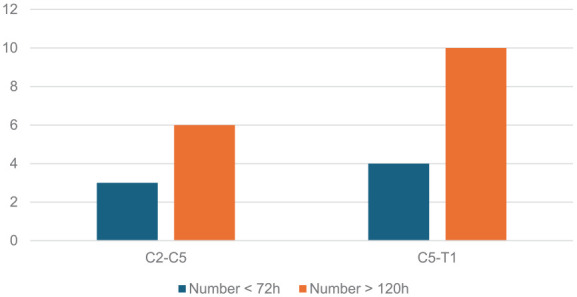
Distribution of SCI level across treatment groups in participants in the trial by Dvorak et al. (2004).^14^

### Risk of bias

Risk of Bias scoring via Downs and Black is shown in Table 3. Due to being at particularly high risk of bias (4/27), we have excluded the study conducted by Kuric et al.^15^ from the narrative synthesis of outcomes.

**Table 3.** Downs and Black scores for risk of bias.

Study	Reporting Bias Score (/10)	External Validity Score (/3)	Internal Validity Score (/7)	Confounding and Power Score (/7)	Overall (/27)
Cinotti et al. (2019)^13^	9	3	4	2	18
Dvorak et al. (2004)^14^	7	3	1	4	15
Kuric et al. (1989)^15^	1	1	0	2	4
Rowan et al. (2004)^16^	3	3	2	3	11

### Narrative synthesis of results of individual studies

#### Change in neurological status

Initiating feed in under 24 h was associated with improvements in neurological status when used as part of a broader bundle of care. Cinotti et al.^13^ were the only group to report this outcome. Earlier initiation of enteral feeding was associated with a statistically significant improvement in ASIA motor score at discharge from ICU (+16 points vs +6 points; *p* < 0.05). After a year, the improvement in ASIA motor score was even more marked in the group which received earlier feeding (33 points difference; *p* < 0.05).

#### Length of stay in ICU

Earlier feeding regimens were not clearly associated with changes in length of ICU admission. The single RCT demonstrated that feeding before 72 h post-injury was associated with a 15.1 day longer stay than starting feeding after 120 h; however no statistical testing was conducted.^14^ Cinotti et al.^13^ found no statistically significant difference between their treatment and control groups. The New Zealand study found that patients who started enteral feeding at an average of 48 h remained for a median of 8.8 days (range 2–36.2 days).^16^

#### Mechanical ventilation

There was no clear indication of differences in length of time mechanically ventilated in association with earlier enteral nutrition. Overall, Cinotti et al.^13^ found no significant difference in the length of time patients spent being mechanically ventilated between the control group and those receiving a specific care bundle (median 15 days vs 17 days; *p* = 0.2). They also found no significant difference in the time to tracheostomy.

While Dvorak et al.^14^ observed that patients who started feeding before 3 days had a longer average mechanical ventilation time (762 h) compared to those after more than 5 days (502 h), no statistical analysis was performed on this difference.

#### Infection

There is no evidence of a change in infection risk with earlier feeding. Cinotti et al.^13^ reported no statistically significant septic complications between groups. Likewise, in the RCT there were equal numbers of septic complications in the group fed early versus the group fed late.

#### Mortality in ICU and at 1 year

There was no evidence of association between mortality and earlier feeding. There was no difference in mortality between the groups in the Cinotti et al.^13^ quasi-experimental study. The mortality rate in ICU in the patient group observed by Rowan et al.^16^ was 2/33 (6.1%).

#### Feeding complications/enteral feed interruptions and rationale

Feeding complications associated with early enteral nutrition were generally low across reporting studies. Dvorak et al.^14^ reported 39 complications in the early feeding group and 59 in the late feeding group. Nausea and excessive diarrhoea were more common in the late group, while early feeding saw a higher use of prokinetics due to intolerance.

Rowan et al.^16^ also noted low overall feeding complication rates, with an average of 2 days of feeding interruption per patient; this was mostly due to high gastric aspirates (>100–200 ml), vomiting, tube issues and ileus. Most patients experienced fewer than four feed interruptions overall.

## Discussion

This systematic review highlights an interplay between timing of enteral feeding and clinical outcomes in SCI patients which is challenging to interpret, with many equivocal results from low quality studies that are all prone to bias.

There is possible benefit in initiating feeding within 24 h to improve neurological recovery, however the study this comes from was confounded by the fact earlier feeding was used as part of a broader bundle of care in that trial.

The impact of earlier feeding on other key outcomes, such as ICU length of stay, duration of mechanical ventilation, infection risk and mortality, is unclear. While delayed feeding in some studies showed a signal indicating shorter ICU stays and reduced ventilation times, the difference was not statistically significant. No difference in infection or mortality was observed between early and delayed feeding. This suggests that early enteral nutrition may be safe.

Feeding complications were generally low, with a possible increase in feed intolerance seen in those fed earlier. However, the 100–200 ml volume used to define feed intolerance in the reporting study is a very conservative volume when one considers that aspirates as high as 500 ml have been shown to not lead to any increased risk of adverse events in general ICU patients. It is also the volume currently recommended by ESPEN guidance to contribute to the multimodal assessment of feed intolerance.^10^

### Limitations

The evidence included in this review is of low quality. It is challenging to determine true effect size of earlier feeding; only one study recorded objective diagnoses of spinal cord injury level as inclusion criteria. Across the included studies, there was no overt control for comorbid injury status amongst many other confounders. There was no time-series based analysis when measuring most outcomes.

The review process itself may be limited by only using conventional databases for medical research. We did not use Scopus or Web of Science, and excluded non-English publications (although this was limited to a single text after title and abstract screening). Similarly, our decision to exclude conference posters and abstracts may have reduce the amount of data available to us. We are however confident that our review has found appropriate peer-reviewed data due to covering the same papers as have been covered in prior reviews on this topic.^17,18^

### Future practice, policy and research

A finding that was consistent across all three studies in the narrative synthesis was uniformity in approach to feeding regimen, which may not be optimal. Substantial heterogeneity in caloric expenditure was shown by Rowan and Kazemi^19^ in a 2020 study with some SCI patients having a difference of almost 1500 kcal in terms of energy expenditure. There is therefore a risk of both under and overfeeding without accurate calculation, both of which would be detrimental to patient outcomes.

There may therefore need to be a change in the titration of caloric intake for these patients, with use of indirect calorimetry as opposed to formulae such as the Harris-Benedict equation. Thus, in future we may need to look at conducting a multicentre study of titration based on indirect calorimetry with Harris-Benedict or current ESPEN guidelines as a control, in conjunction with wider implementation of early feeding regimens. Single specialist centres would not be able to admit enough patients to conduct a trial with enough statistical power, therefore any trial would need to have a multicentre basis.

## Conclusion

There is very low-quality evidence that early enteral feeding (initiated less than 72 h into an ICU admission) is as safe as delayed feeding in patients with acute spinal cord injury. There is insufficient evidence to determine that earlier feeding benefits long term neurological outcome. Whilst the metabolic effects of spinal cord injury are known to be different to other traumatic injuries, in the absence of significant evidence of harm, early nutrition is likely to be safe with monitoring for complications.

## Appendix 1

### Medline via Ovid 12th February 2023 (DATES COVERED)

1. (feeding tube* or PEG line* or EN or enteral nutrition or enteric nutrition or enteric feeding or enteral feed* or enteric feed*).ti,ab.
2. (NG or nasogastric).ti,ab.
3. Enteral Nutrition/
4. (NJ or nasojejunal).ti,ab.
5. 1 or 2 or 3 or 4
6. exp Spinal Cord Injuries/
7. exp Spinal Cord Ischemia/
8. (myelopathy and trauma*).ti,ab.
9. ((Spin* or cord) and (Contusion or lacerat* or transect* or trauma* or ischaemi*)).ti,ab.
10. (SCI or aSCI or (Acute and SCI)).ti,ab.
11. exp Paraplegia/
12. exp Quadriplegia/
13. (paraplegia* or quadriplegia* or tetraplegia*).ab,ti.
14. 6 or 7 or 8 or 9 or 10 or 11 or 12 or 13
15. Critical Care/
16. Critical Illness/
17. Intensive Care Units/
18. (intensiv* or critical* or emerg* or acu*).ti,ab.
19. 15 or 16 or 17 or 18
20. 5 and 14 and 19

### EMBASE 12th February 2023

1. (feeding tube* or PEG line* or EN or enteral nutrition or enteric nutrition or enteric feeding or enteral feed* or enteric feed*).ti,ab.
2. (NG or nasogastric).ti,ab.
3. exp enteric feeding/
4. (NJ or nasojejunal).ti,ab.
5. 1 or 2 or 3 or 4
6. exp spinal cord injury/
7. exp spinal cord ischemia/
8. (myelopathy and trauma*).ti,ab.
9. ((spin* or cord) and (Contusion or lacerat* or transect* or trauma* or ischaemi*)).ti,ab.
10. (SCI or aSCI or (Acute and SCI)).ti,ab.
11. (spinal cord adj2 injur*).ti,ab.
12. exp paraplegia/
13. exp quadriplegia/
14. (paraplegi* or quadriplegi* or tetraplegi*).ti,ab.
15. 6 or 7 or 8 or 9 or 10 or 11 or 12 or 13 or 14
16. exp intensive care/
17. exp critical illness/
18. exp intensive care unit/
19. (intensiv* or critic* or emerg* or acu*).ti,ab.
20. 16 or 17 or 18 or 19
21. 5 and 15 and 20

### Cochrance CENTRAL Trials

#1 MeSH descriptor: [Enteral Nutrition] explode all trees
#2 (feeding tube* or PEG line* or EN or enteral nutrition or enteric nutrition or enteric feeding or enteral feed* or enteric feed*)
#3 NG or nasogastric
#4 NJ or nasojejunal
#5 #1 OR #2 OR #3 OR #4
#6 MeSH descriptor: [Spinal Cord Injuries] explode all trees
#7 spin* near/2 (fracture* or wound* or trauma* or injur* or damag*)
#8 aSCI or SCI or spinal cord injur*
#9 MeSH descriptor: [Paraplegia] explode all trees
#10 MeSH descriptor: [Quadriplegia] explode all trees
#11 paraplegi* or quadriplegi* or tetraplegi*
#12 #6 #7 OR #8 OR #9 OR #10 OR #11
#13 MeSH descriptor: [Intensive Care Units] explode all trees
#14 MeSH descriptor: [Critical Care] explode all trees
#15 MeSH descriptor: [Critical Illness] explode all trees
#16 (intensiv* or critical* or hyperacute* or acute*) and (care or unit* or nurs*)
#17 #13 OR #14 OR #15 OR #16
#18 #5 AND #12 AND #17

## References

[1] Patek M Stewart M. Spinal cord injury. Anaesth Intensive Care Med 2020; 21: 411–416.

[2] Kang Y Ding H Zhou H , et al. Epidemiology of worldwide spinal cord injury: a literature review. J Neurorestoratology 2018; 6: 1–9.

[3] Bellamy R Pitts FW Stauffer ES. Respiratory complications in traumatic quadriplegia. Analysis of 20 years’ experience. J Neurosurg 1973; 39: 596–600. 4743566 10.3171/jns.1973.39.5.0596

[4] Gilbert J. Critical care management of the patient with acute spinal cord injury. Crit Care Clin 1987; 3: 549–567. 3332214

[5] Multidisciplinary Association of Spinal Cord Injury Professionals. Management of neurogenic bowel dysfunction. Stoke Mandeville Hospital: MASCIP Guidelines, 2021, p.46.

[6] Cederholm T Barazzoni R Austin P , et al. ESPEN guidelines on definitions and terminology of clinical nutrition. Clin Nutr 2017; 36: 49–64. 27642056 10.1016/j.clnu.2016.09.004

[7] Keel M Trentz O. Pathophysiology of polytrauma. Injury 2005; 36: 691–709. 15910820 10.1016/j.injury.2004.12.037

[8] Chiarelli A Enzi G Casadei A , et al. Very early nutrition supplementation in burned patients. Am J Clin Nutr 1990; 51: 1035–1039. 2112339 10.1093/ajcn/51.6.1035

[9] Doig GS Heighes PT Simpson F , et al. Early enteral nutrition, provided within 24 h of injury or intensive care unit admission, significantly reduces mortality in critically ill patients: a meta-analysis of randomised controlled trials. Intensive Care Med 2009; 35: 2018–2027. 19777207 10.1007/s00134-009-1664-4

[10] Singer P Blaser AR Berger MM , et al. ESPEN guideline on clinical nutrition in the intensive care unit. Clin Nutr 2019; 38: 48–79. 30348463 10.1016/j.clnu.2018.08.037

[11] Page MJ McKenzie JE Bossuyt PM , et al. The PRISMA 2020 statement: an updated guideline for reporting systematic reviews. BMJ 2021; 372: n71. 10.1136/bmj.n71 PMC8005924 33782057

[12] Downs SH Black N. The feasibility of creating a checklist for the assessment of the methodological quality both of randomised and non-randomised studies of health care interventions. J Epidemiol Community Health 1998; 52: 377–384. 9764259 10.1136/jech.52.6.377 PMC1756728

[13] Cinotti R Demeure-Dit-Latte D Mahe PJ , et al. Impact of a quality improvement program on the neurological outcome of patients with traumatic spinal cord injury: a before-after mono-centric study. J Neurotrauma 2019; 36: 3338–3346. 30907244 10.1089/neu.2018.6298

[14] Dvorak MF Noonan VK Bélanger L , et al. Early versus late enteral feeding in patients with acute cervical spinal cord injury: a pilot study. Spine 2004; 29: E175–E180. 10.1097/00007632-200405010-00020 15105682

[15] Kuric J Lucas CE Ledgerwood AM , et al. Nutritional support: a prophylaxis against stress bleeding after spinal cord injury. Paraplegia 1989; 27: 140–145. 2497427 10.1038/sc.1989.21

[16] Rowan CJ Gillanders LK Paice RL , et al. Is early enteral feeding safe in patients who have suffered spinal cord injury? Injury 2004; 35: 238–242. 15124789 10.1016/s0020-1383(03)00203-1

[17] Reintam Blaser A Starkopf J Alhazzani W , et al. Early enteral nutrition in critically ill patients: ESICM clinical practice guidelines. Intensive Care Med 2017; 43: 380–398. 28168570 10.1007/s00134-016-4665-0 PMC5323492

[18] Thibault-Halman G Casha S Singer S , et al. Acute management of nutritional demands after spinal cord injury. J Neurotrauma 2011; 28: 1497–1507. 20373845 10.1089/neu.2009.1155 PMC3143385

[19] Rowan C Kazemi A. An observational study of feeding practice in ventilated patients with spinal cord injury. Clin Nutr ESPEN 2020; 37: 107–113. 32359731 10.1016/j.clnesp.2020.03.010

